# Genetic diversity and distribution pattern responses of *Picea asperata*

**DOI:** 10.3389/fpls.2026.1808288

**Published:** 2026-05-28

**Authors:** Qinyuan Chen, Xin Tang, Yu Yin, Dafu Ru, Shuo Feng

**Affiliations:** 1 State Key Laboratory of Plateau Ecology and Agriculture, Qinghai University, Xining, China; 2 State Key Laboratory of Herbage Improvement and Grassland Agro-Ecosystems, College of Ecology, Lanzhou University, Lanzhou, China

**Keywords:** genetic diversity, *Picea asperata*, population structure, redundancy analysis, species distribution modeling

## Abstract

**Introduction:**

*Picea asperata* Mast. as a kind of keystone coniferous plant, lives in alpine and subalpine regions of Southwest China and takes on very important ecological and economic tasks there. But with the increasingly serious global climate events, the living environment for *P. asperata* is getting into great trouble. In this situation, explaining the inherent connection between species distribution and environmental elements is a main part of ecological study. This kind of understanding not only can help us to know about the genetic variety, but also helps us to think about the variety of living organisms.

**Methods:**

We employed transcriptome sequencing to analyze 54 P*. asperata* individuals from 11 natural populations and combined with environmental variables to assess population structure and genetic diversity. Species distribution modeling (MaxEnt) was applied to predict habitat shifts under current and future climate scenarios (SSP1-2.6, SSP5-8.5), while redundancy analysis (RDA) and latent factor mixed model (LFMM) identified key environmental factors that drive genetic variation.

**Results:**

ADMIXTURE analysis indicated that the optimal number of clusters was K = 1, and *P. asperata* exhibited low nucleotide diversity (*π* = 0.003508). Stairway Plot analysis revealed a historical bottleneck followed by gradual population recovery. TreeMix analysis indicated ongoing gene flow among populations, consistent with low pairwise *F*_ST_ values. MaxEnt projections predicted an expansion of suitable habitats under future climates, particularly under the high−emission SSP5−8.5 scenario. Both RDA and LFMM identified December wind speed (Wind12) and precipitation of the warmest quarter (Bio18) as the environmental factors most strongly associated with genomic differentiation. Functional annotation and GO enrichment further uncovered candidate genes involved in stress responses, including *ERD1*, *ARR1*, and *IBR1*.

**Conclusions:**

The study reveals low genetic diversity in *P. asperata*. Stairway Plot analysis detected a bottleneck during the Quaternary glaciations. MaxEnt projections indicate habitat expansion under future climates, while GEA analyses identify December wind speed (Wind12) and the warmest quarter (Bio18) as key drivers of genomic differentiation. To safeguard the adaptive potential of *P. asperata*, we recommend strengthening *in situ* conservation, maintaining existing habitat connectivity, assisting the migration of germplasm carrying key adaptive alleles, and establishing comprehensive germplasm repositories.

## 1 Introduction

Global climate change can be referred to as one of the biggest environmental issues that the world will come across in the 21st century. It affects the ecosystems and biodiversity all around the world. The industrial revolution has caused a large amount of greenhouse gas emissions and has caused the earth’s temperature to increase more rapidly (Ramanathan and Feng, 2009), and extreme weather events have also happened more frequently, and the pattern of precipitation has also changed. This will affect the whole of the Earth’s climate system and species distribution, population, and ecosystems (Copenhaver‐Parry et al., 2020; Compagnoni et al., 2021). So many species are under the threat of losing their homes, having smaller areas in which to live, and dying because it is getting warmer and raining differently (Jones et al., 2016). The effect of climate change on biodiversity happens at different levels in an ecosystem. At the ecosystem level, with the increase in temperature, mountain vegetation zones will rise, and the habitat fragmentation intensified by the upward movement of the vegetation zones (Hamid et al., 2020), which again leads to decrease of the species interaction among species in an ecosystem and ultimately leads to lower resilience in an ecosystem (Davidson, 2014; Barredo et al., 2020). And then it flows on to species diversity, because a lot of organisms won’t be able to track suitable climates, and so they contract ranges and they go up in extinction risk (Bellard et al., 2012). Habitat loss and population fragmentation caused by climate change erode the genetic diversity of the foundation of biodiversity by lowering the effective size of the population, raising the rate of inbreeding, and intensifying genetic drift (Lande, 1988). Erosion like this reduces adaptability, a pattern that studies find in low genetic diversity in forests and the reduced capacity to select for climate in forest trees. For example, the tree species that grow slowly, such as *Picea* species, and these kinds of species have a migration rate less than climate change, genetic diversity now is very important for them to continue evolution.

*Picea asperata* Mast. belongs to the genus *Picea* of the family Pinaceae, and is commonly known as thick-branch spruce, Maoxian spruce, and large fruit spruce. It is an evergreen conifer, which is a prominent species of coniferous tree in southwestern China. It is possible to find this species in all parts of the highland and sub-alpine area in the southwest of China. It has the feature that can endure adversity, with cold resistance, drought-bearing ability, and thus it will necessarily have to play a certain role in terms of protecting the high mountain ecosystem and advancing ecological greening works. *P. asperata* provides certain ecological service benefits to the local ecosystems, such as soil and water conservation, carbon sequestration, and economic services in production and forestry. With the global climate change increasingly fiercer, the habitat of *P. asperata* faces greater burdens, thus this will cause the change phenomenon of the distribution as well as the pattern of change of the population (Naudiyal et al., 2021). Then, in order to improve the effectiveness of the protection strategy, studying the population structure, genetic diversity and how *P. asperata* responds to future climate change is also needed. In the past, research on *P. asperata* mainly focused on disease prevention and the management of plantation forests, and the research on the population structure of its individuals and the shift of its future distribution is relatively scarce.

In the past few years, with the development of high-throughput sequencing technology, the study of population genetic diversity and adaptation has been promoted. Compared with traditional technology, it is obvious that high-throughput sequencing technology has a distinct advantage of large-scale parallel sequencing. At the same time, millions of DNA molecules or even billions can be sequenced at once. Sequence throughput and sequencing accuracy are dramatically improved compared with the traditional method. Reversible terminator sequencing new technologies such as have made sequence-by-synthesis a reality, which allows for complete understanding of complex genomes and discovering genotype-environment relationships and precise identification of the variant gene and adaptation. Furthermore, with the decrease in the cost of high-throughput sequencing, it has been widely applied in different fields such as genomics, transcriptomics and proteomics (Wang, 2023). While much of the research on *P. asperata* in previous works has focused on disease resistance and plantation management (Zhao et al., 2022; Tao et al., 2024; Liu et al., 2025), transcriptome-based research on the genetic diversity of *P. asperata* is still scarce. This study has done some transcriptome sequencing work on *P. asperata* in order to study population genetic structure, adaptive reactions of *P. asperata* facing different environments, and provides an effective way of maintaining the diversity.

In this study, we analyzed 54 individual samples collected from 11 natural populations of *P. asperata*. Based on transcriptome sequencing, we performed population genomic and landscape genomic analyses. The aims of this study were (i) to assess genome-wide genetic diversity, (ii) to identify environmental and geographical variables that drive genetic differentiation, while identifying key environmental variables that affect the growth of *P. asperata*, and (iii) to predict the potential changes in the distribution range of *P. asperata* under the background of future climate change. These analyses aim to provide scientific strategies for the future conservation work of *P. asperata*.

## 2 Materials and methods

### 2.1 Sample collection and data preparation

**Figure 1:**
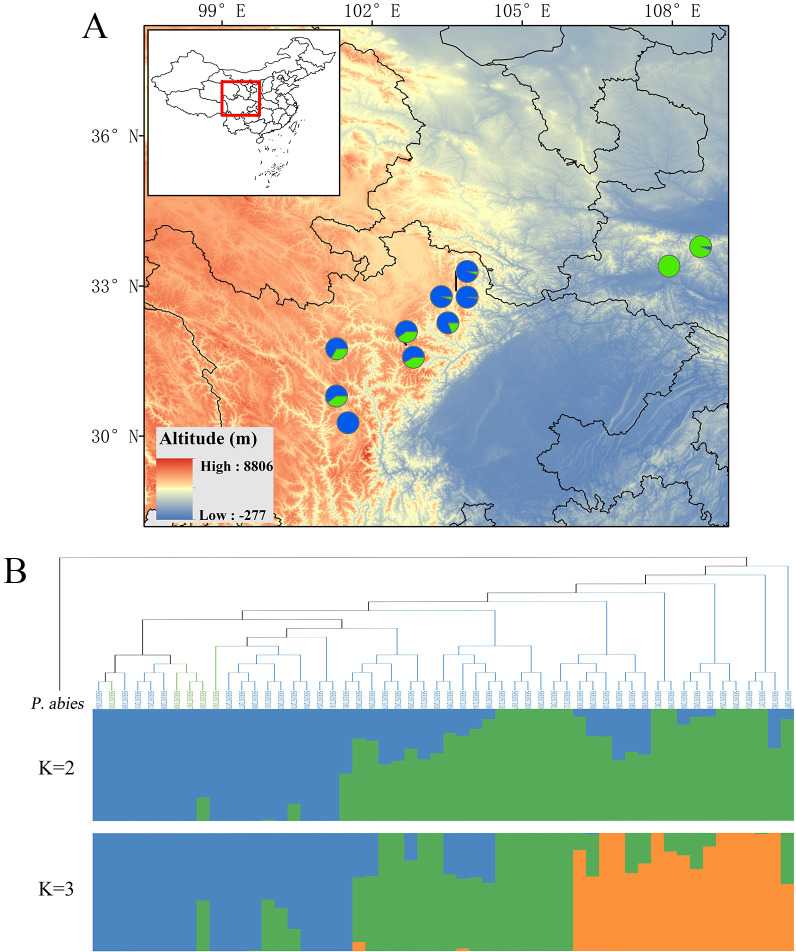
Population genetic analyses of all sampled *Picea asperata* individuals. **(A)** Geographical distribution of *P. asperata* populations, with colors in the pie charts referring to genetic groups identified in an admixture analysis when K = 2. **(B)** Genetic structure and phylogenetic tree of the *P. asperata* populations.

The sampling for this study was mainly conducted in the eastern Qinghai-Tibet Plateau. Guided by existing findings and in accordance with the Flora of China and relevant literature, we documented and collected *P. asperata* population samples. Subsequent population structure analysis based on sequencing data, along with population genetic and phylogenetic analyses, indicated that sampled individuals from Gansu and Qinghai provinces genetically belong to *P. crassifolia* (Feng et al., 2023a). To ensure the accuracy of our study, only samples from natural populations located in Sichuan and Shaanxi were retained. Collect samples of fresh mature needles from first-year branches from 11 natural populations of *P. asperata*, with individuals within each population spaced at least 100 meters apart. All materials were collected from fresh needles facing the sun in July and August. We obtained 54 individuals from 11 natural populations (**Figure 1A**; **Table 1**). The distribution map (**Figure 1A**) was downloaded from the DataV.GeoAtlas (https://datav.aliyun.com/portal/school/atlas/area_selector) and converted from JSON format to SHP format in MapShaper (https://mapshaper.org/), finally constructed using ArcGIS v10.8.2. Immediately freeze and store the collected needles in liquid nitrogen for the extraction of total RNA.

**Table 1:** Population locations and sample sizes of *Picea asperata*.

Population	Number of individuals	Location	Longitude (E)	Latitude (N)	Altitude (m)
Asp01	2	Kangding City, Sichuan Province	101.5174	30.2668	3517.88
Asp02	5	Daofu County, Sichuan Province	101.2875	30.8001	3645.75
Asp03	9	Rangtang County, Sichuan Province	101.2897	31.7478	2947.12
Asp04	4	Li County, Sichuan Province	102.8243	31.5762	2789.47
Asp05	7	Li County, Sichuan Province	102.6855	31.8219	3269.94
Asp06	8	Songpan County, Sichuan Province	103.5148	32.3774	2666.39
Asp07	8	Songpan County, Sichuan Province	103.8978	32.7782	2702.82
Asp08	3	Songpan County, Sichuan Province	103.5597	32.7970	3320.00
Asp09	3	Songpan County, Sichuan Province	103.6778	32.9078	3230.00
Asp11	1	Foping County, Shaanxi Province	107.9351	33.3973	1117.00
Asp12	4	Xi’an City, Shaanxi Province	108.5672	33.7871	1502.00

Total RNA was extracted from *P. asperata* needles using an improved CTAB method. RNA concentration was assessed using an Agilent 2100 Bioanalyzer (Ma et al., 2015; Rahmani and Amraee, 2020). After passing quality control, RNA libraries were constructed. Qualified libraries were denatured with NaOH to generate single-stranded DNA, then diluted to an appropriate concentration according to the desired sequencing depth. The denatured and diluted library was loaded onto a FlowCell, where it hybridized with adapter sequences on the FlowCell. Bridge PCR amplification was performed on the cBot platform to generate clusters. Finally, the prepared FlowCell was sequenced using the Illumina HiSeq 2500 platform (Illumina Inc., San Diego, CA, USA) in paired-end mode, ensuring a minimum sequencing data volume of 6 Gb per individual.

Raw reads were trimmed with fastp v0.24.0 (Chen et al., 2018) using the parameters: -f 10 -F 10 -x -g -c -q 15 -u 40 -n 5. The filtered paired-end sequencing data were aligned to the *P. abies* reference transcriptome (Nystedt et al., 2013; Delhomme et al., 2015; Ru et al., 2016; Feng et al., 2023a, Feng et al, 2023b) using HISAT2 v2.2.1 (Kim et al., 2019), generating SAM files that were subsequently converted to sorted and indexed BAM format using SAMtools v1.21 (Li et al., 2009). PCR duplicates were marked and removed with GATK v4.4 MarkDuplicates (DePristo et al., 2011), and read group information was appended using Picard. Indel realignment was performed with GATK v3.8 to correct alignment errors caused by indels.

SNP calling was performed on the BAM files using SAMtools v1.21 and BCFtools v1.21 (Danecek et al., 2021), generating raw VCF files. Custom Perl scripts were employed to filter the VCF files, removing low-depth (DP < 10) and low-quality (QUAL < 30) sites, as well as SNPs within 5 bp of Indels to minimize false-positive variants. Merging of multi-sample VCF files was conducted using BCFtools v1.21, followed by further filtering with VCFtools v0.1.16 (Danecek et al., 2011). The final VCF file is obtained by removing high missing rate sites (max-missing-count 5) and non-biallelic sites (min-alleles 2, max-alleles 2), while retaining sites with non-reference allele frequencies greater than 1% (non-ref-af 0.01) for downstream analysis.

### 2.2 Population structure, phylogenomics and genetic diversity

The filtered VCF file was converted to FASTA format using a Perl script, and sequences were aligned with MAFFT v7.525 (Katoh and Standley, 2013). A maximum likelihood (ML) phylogenetic tree reflecting the evolutionary relationships of *P. asperata* populations was constructed using IQ-TREE v2.3.6 (Nguyen et al., 2015) for the FASTA files that completed the alignment. Referring to the tree building methods of Wang et al (Wang et al., 2025), a GTR+F+R6 model was used, with *P. abies* as the outgroup and a bootstrap value of 1,000. At the same time, principal component analysis (PCA) was performed using PLINK v1.9 (Purcell et al., 2007), and population structure analysis was conducted on the samples in ADMIXTURE v1.3.0 (Alexander and Lange, 2011). Cross-validation errors (CV errors) were calculated for different K values (K = 1-10) (**Supplementary Figure 1**) to determine the optimal number of clusters. Visualization was completed in iTOL v7.1 (https://itol.embl.de) and R v4.4.2.

To further understand the genetic diversity of the population, VCFtools v0.1.16 was used to calculate the genetic differentiation (*F*_ST_), nucleotide diversity (*π*), expected heterozygosity (*H*_e_), observed heterozygosity (*H*_o_) and Tajima’s *D*. We used TreeMix v1.13 (Pickrell and Pritchard, 2012) to infer possible gene flow among populations. Migration events (m) were analyzed from 0 to 10. The parameter -noss was used to prevent overcorrection of the standard errors. The migration tree was visualized using the plot_tree function in R v4.1.0.

To investigate the historical population dynamics of *P. asperata*, we inferred the historical effective population size (Ne) trajectories using Stairway Plot v2.2 (Liu and Fu, 2015). The folded site frequency spectrum (SFS) required for Stairway Plot analysis was constructed using easySFS, a tool that implements the conversion of VCF-formatted data to SFS format based on the Spectrum class of the dadi package (Gutenkunst et al., 2009).

### 2.3 Species distribution modeling

To assess the distribution trends of *P. asperata* under current and future climate change scenarios, we conducted species distribution modeling (SDM) based on verified occurrence records. A total of 72 occurrence points were compiled from the Global Biodiversity Information Facility (GBIF, https://www.gbif.org), the Chinese Virtual Herbarium (CVH, https://www.cvh.ac.cn), and field surveys. To prevent the impact of spatial autocorrelation in species distribution data, distribution locations with a distance less than 5 km were removed in ArcGIS v10.8.2, and a total of 60 distribution locations were retained. We used the MaxEnt v3.4.4 (Phillips et al., 2006) to predict the potential distributions of all individuals with the maximum entropy method, current and future in four time periods: the 2030s (2021-2040), the 2050s (2041-2060), the 2070s (2061-2080), and the 2090s (2081-2100). Future climate projections were derived from the Sixth Phase of the Coupled Model Intercomparison Project (CMIP6), incorporating four Shared Socioeconomic Pathways (SSPs) representing different greenhouse gas emission scenarios: SSP1-2.6, SSP2-4.5, SSP3-7.0, and SSP5-8.5. For this study, we focused on the SSP1−2.6 (low emissions) and SSP5−8.5 (high emissions) scenarios. Current and future bioclimatic data were downloaded at a 30−second spatial resolution from WorldClim v2.1 (https://www.worldclim.org).

The future climate models included only 19 bioclimatic variables (**Supplementary Table 1**). For current and future niche modeling, eight environmental factors (Bio3: Isothermality, Bio4: Temperature Seasonality, Bio7: Temperature Annual Range, Bio10: Mean Temperature of Warmest Quarter, Bio12: Annual Precipitation, Bio13: Precipitation of Wettest Month, Bio14: Precipitation of Driest Month, Bio15: Precipitation Seasonality) were selected based on Pearson correlation coefficients (r) calculated using IBM SPSS Statistics v26.0. In ArcGIS v10.8.2, the distribution areas were classified into unsuitable area, lowly suitable area, moderately suitable area, and highly suitable area using the natural breaks method. Model performance was evaluated using the True Skill Statistic (TSS), calculated as TSS = Sensitivity + Specificity – 1, where sensitivity and specificity were derived from the MaxEnt output under the maximum training sensitivity plus specificity threshold.

### 2.4 Redundancy analysis

To disentangle the associations between genomic differentiation and environmental variation, we implemented two complementary genotype-environment association (GEA) studies. The filtered VCF file was converted to the format needed for redundancy analysis (RDA) using VCFtools v0.1.16 and PLINK v1.9 (Purcell et al., 2007). To ensure comprehensive coverage of potentially relevant climatic and topographic dimensions that might influence the genetic variation of *P. asperata*, we downloaded 68 environmental variables (**Supplementary Table 1**) from WorldClim v2.1 (https://www.worldclim.org) at a 30-second resolution. To alleviate multicollinearity, pairwise Pearson correlation coefficients (*r*) between each of the variables were calculated using IBM SPSS Statistics v26.0, and one variable from each pair with |*r*| > 0.7 was deleted. Subsequently, variance inflation factor (VIF) analysis was done to further assess multicollinearity among the remaining variables. This two−step screening ensured that the selected predictors were not highly correlated, thereby avoiding bias in the subsequent RDA. Following this screening, five environmental factors (Bio12: Annual Precipitation, Bio18: Precipitation of Warmest Quarter, Srad5: May Solar Radiation, Wind12: December Wind Speed, Alt: Altitude) were selected for subsequent analysis. Redundancy analysis and latent factor mixed model (LFMM) were performed using the VEGAN package and LFMM package in R v4.4.2 to evaluate linear relationships between SNP data and environmental variables and the overlap between SNPs detected by the two methods was examined, then the results were visualized for interpretation.

After mapping the candidate SNPs shared by RDA and LFMM to the reference transcriptome of *P. abies*, we performed functional annotation using EggNOG-mapper v2.1.4 (Cantalapiedra et al., 2021) with an E-value threshold of 1.0×10^-3^. Subsequently, Gene Ontology (GO) enrichment analysis was conducted using TBtools v2.441 (Chen et al., 2023), and *p*-values were corrected by the Benjamini-Hochberg false discovery rate (FDR) method to explore the functions of these genes (Benjamini and Hochberg, 1995). Visualization was carried out using the R package ggplot2. Finally, genes related to environmental adaptation identified from GO enrichment were further screened and aligned to the *Arabidopsis thaliana* proteome using BLAST.

## 3 Results

54 samples were sequenced using the Illumina HiSeq 2500 platform, resulting in raw data of 376.09 Gb, with an average of 6.96 Gb per individual. After filtering the raw data, 346.57 Gb of clean data was obtained, and the data results were statistically analyzed (**Supplementary Table 2**). Using *P. abies* as the reference transcriptome, stringent filtering of initial variant calls yielded 35,166 high-quality SNPs.

### 3.1 Population structure and genetic diversity

ADMIXTURE analysis of the population structure of *P. asperata* revealed that the cross−validation (CV) error was lowest at K = 1 (CV_1_ = 0.13682). The phylogenetic tree and PCA were also used to validate the relationships shown by ADMIXTURE, which confirmed genetic clustering patterns for clustering (**Figure 1B**; **Supplementary Figure 2**). We estimated multiple genetic variation parameters (**Table 2**; **Supplementary Tables 3**, **4**). Nucleotide diversity (*π*) was 0.003508, and Tajima’s *D* was 0.488912 (**Table 2**). The TreeMix results show that there is sustained gene flow between populations. This result directly supports the low pairwise *F*_ST_ values (**Supplementary Table 3**).

**Table 2:** Genetic diversity parameters of *Picea asperata*.

Parameter	*Picea asperata*
*π*	0.003508
Tajima’s *D*	0.488912

**Figure 2:**
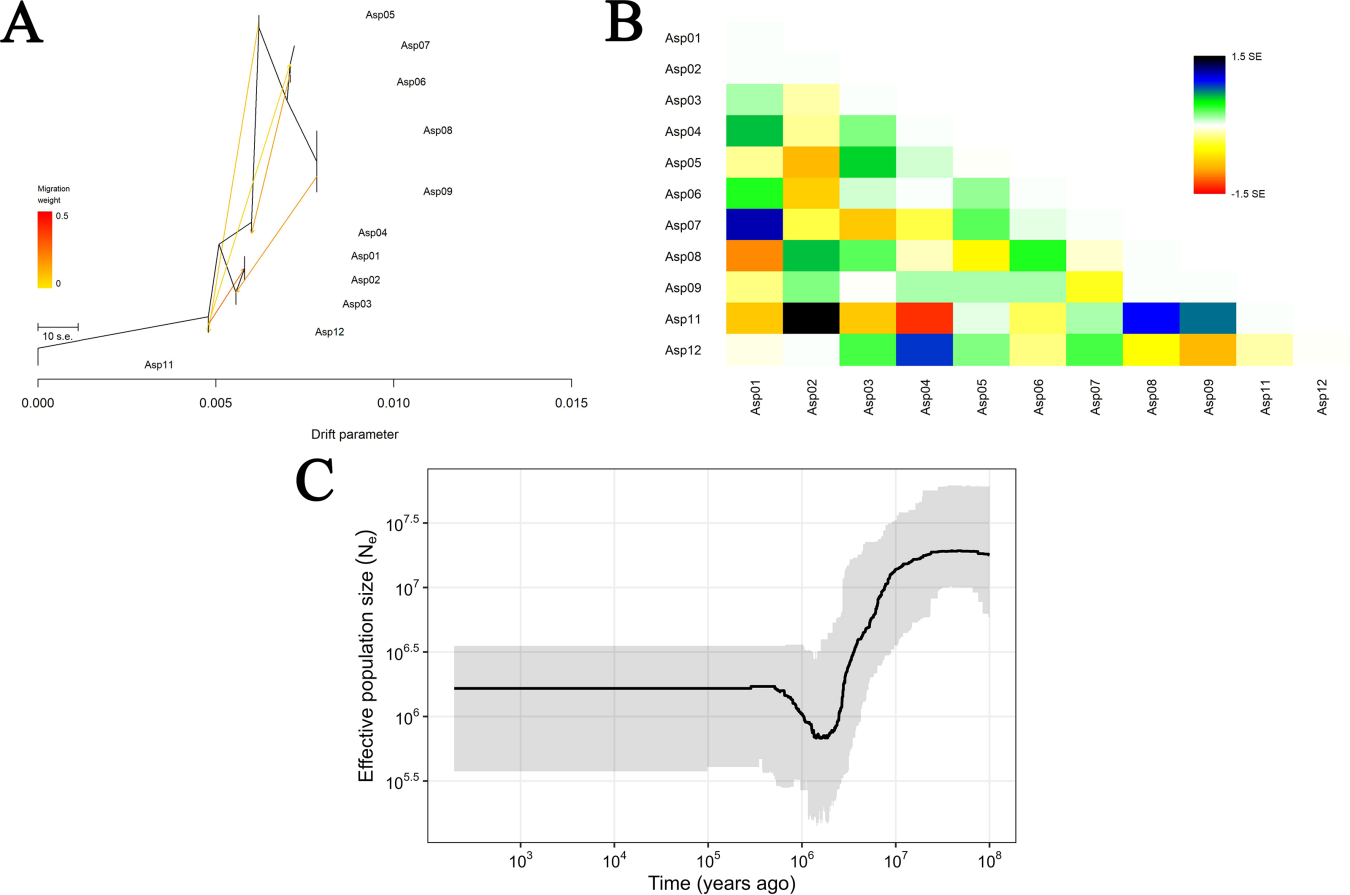
Demographic history and gene flow analyses of *Picea asperata*. **(A)**Maximum likelihood (ML) tree inferred using TreeMix. **(B)** Heatmaps of residual fit from the ML tree. **(C)** Stairway Plot analysis of historical effective population size (Ne) dynamics for *Picea asperata*. Gray shading represents the 95% confidence interval, and the black lines represent the median values.

Based on SNPs from all 54 individuals, the Stairway Plot was used to infer the historical effective population size (Ne) dynamics of *P. asperata*. The results revealed a pronounced bottleneck event occurring at approximately 1.60 million years ago (Ma), during which Ne contracted to 6.76×10^5^ (**Figure 2C**). Following this bottleneck, Ne exhibited a continuous increasing trend, indicating gradual population recovery and expansion after the historical contraction. At present, the *P. asperata* population has reached a stable demographic equilibrium, with a median effective population size maintained at 1.65×10^6^.

### 3.2 Potential distribution forecast

**Figure 3:**
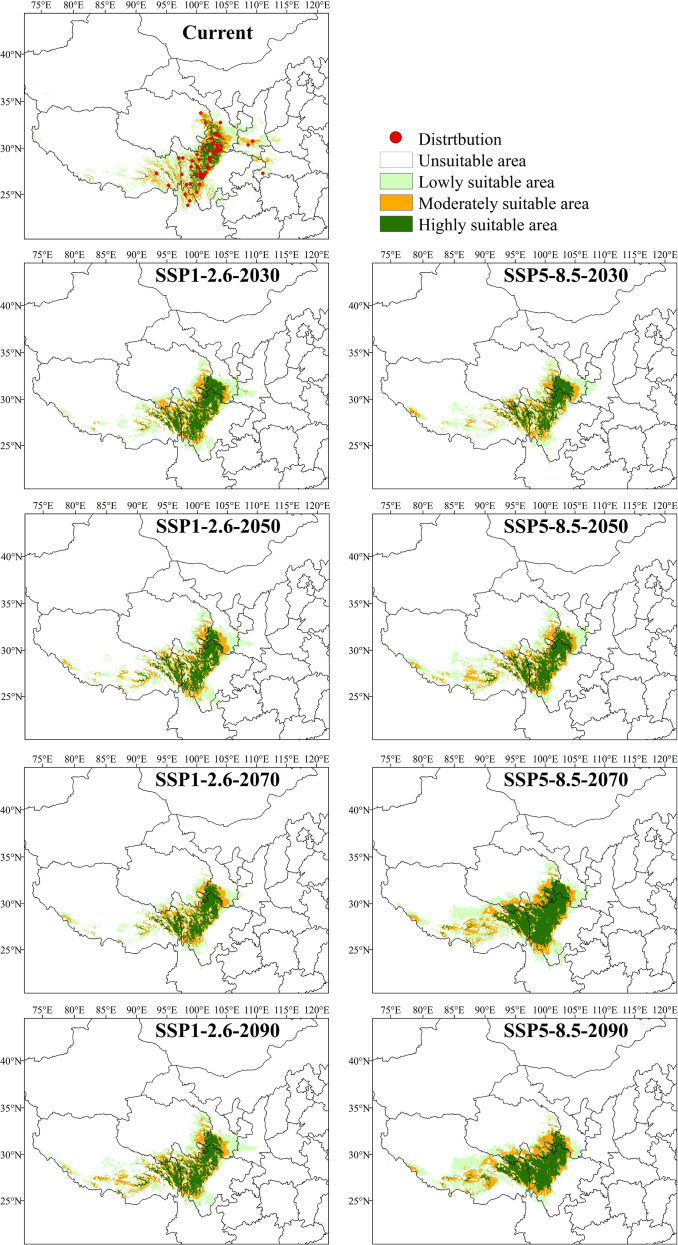
Distribution of *Picea asperata* under current climate conditions and predicted suitable areas for the 2030s–2090s under SSP1−2.6 and SSP5−8.5 scenarios.

In the species distribution model, all MaxEnt models obtained the area under the receiver operating characteristic curve (AUC) values above 0.9 (**Table 3**), indicating that all the models have a high predictive accuracy and reliability in showing the potential distribution pattern of *P. asperata*. The true skill statistic (TSS) values were all greater than 0.86, further confirming the excellent discriminatory ability of the models (**Table 3**). Under the current climatic conditions, the model predictions showed that nearly all actual occurrence points fell within the predicted suitable habitats, predominantly in highly suitable areas, validating a strong consistency between the model predictions results and the actual distribution status. The total current suitable area was estimated at 504,579 square kilometers (**Figure 3**).

**Table 3:** The area, AUC and TSS value of the distribution range of suitable growth areas for *Picea asperata* in each period in the MaxEnt model.

Period	Lowly suitable area	Moderately suitable area	Highly suitable area	Distribution area	AUC	TSS
Current	30.83×10^4^ km^2^	12.49×10^4^ km^2^	7.14×10^4^ km^2^	50.46×10^4^ km^2^	0.993	0.920
SSP1-2.6-2030	28.62×10^4^ km^2^	16.93×10^4^ km^2^	20.28×10^4^ km^2^	65.83×10^4^ km^2^	0.992	0.872
SSP1-2.6-2050	31.17×10^4^ km^2^	17.30×10^4^ km^2^	18.35×10^4^ km^2^	66.82×10^4^ km^2^	0.992	0.865
SSP1-2.6-2070	22.90×10^4^ km^2^	15.14×10^4^ km^2^	15.92×10^4^ km^2^	53.96×10^4^ km^2^	0.994	0.872
SSP1-2.6-2090	35.39×10^4^ km^2^	19.78×10^4^ km^2^	20.46×10^4^ km^2^	75.62×10^4^ km^2^	0.991	0.865
SSP5-8.5-2030	31.44×10^4^ km^2^	16.62×10^4^ km^2^	16.30×10^4^ km^2^	64.36×10^4^ km^2^	0.991	0.944
SSP5-8.5-2050	34.94×10^4^ km^2^	18.87×10^4^ km^2^	19.37×10^4^ km^2^	73.18×10^4^ km^2^	0.990	0.942
SSP5-8.5-2070	37.65×10^4^ km^2^	21.00×10^4^ km^2^	30.69×10^4^ km^2^	89.34×10^4^ km^2^	0.991	0.947
SSP5-8.5-2090	31.83×10^4^ km^2^	21.39×10^4^ km^2^	26.02×10^4^ km^2^	79.24×10^4^ km^2^	0.992	0.948

Predictive analysis of potential distribution under different climate scenarios in the future shows that the suitable area of *P. asperata* will exhibit a dynamic trend of change (**Figure 3**). In the SSP1-2.6 scenario (low emission scenario) and SSP5-8.5 scenario (high emission scenario), the area of suitable habitats expands to 756,247 square kilometers in SSP1-2.6-2090 and 792,394 square kilometers in SSP5-8.5-2090, showing an overall upward trend, especially in highly suitable areas where the expansion trend is more significant.

### 3.3 Association between genomic differentiation and environmental factors

**Figure 4:**
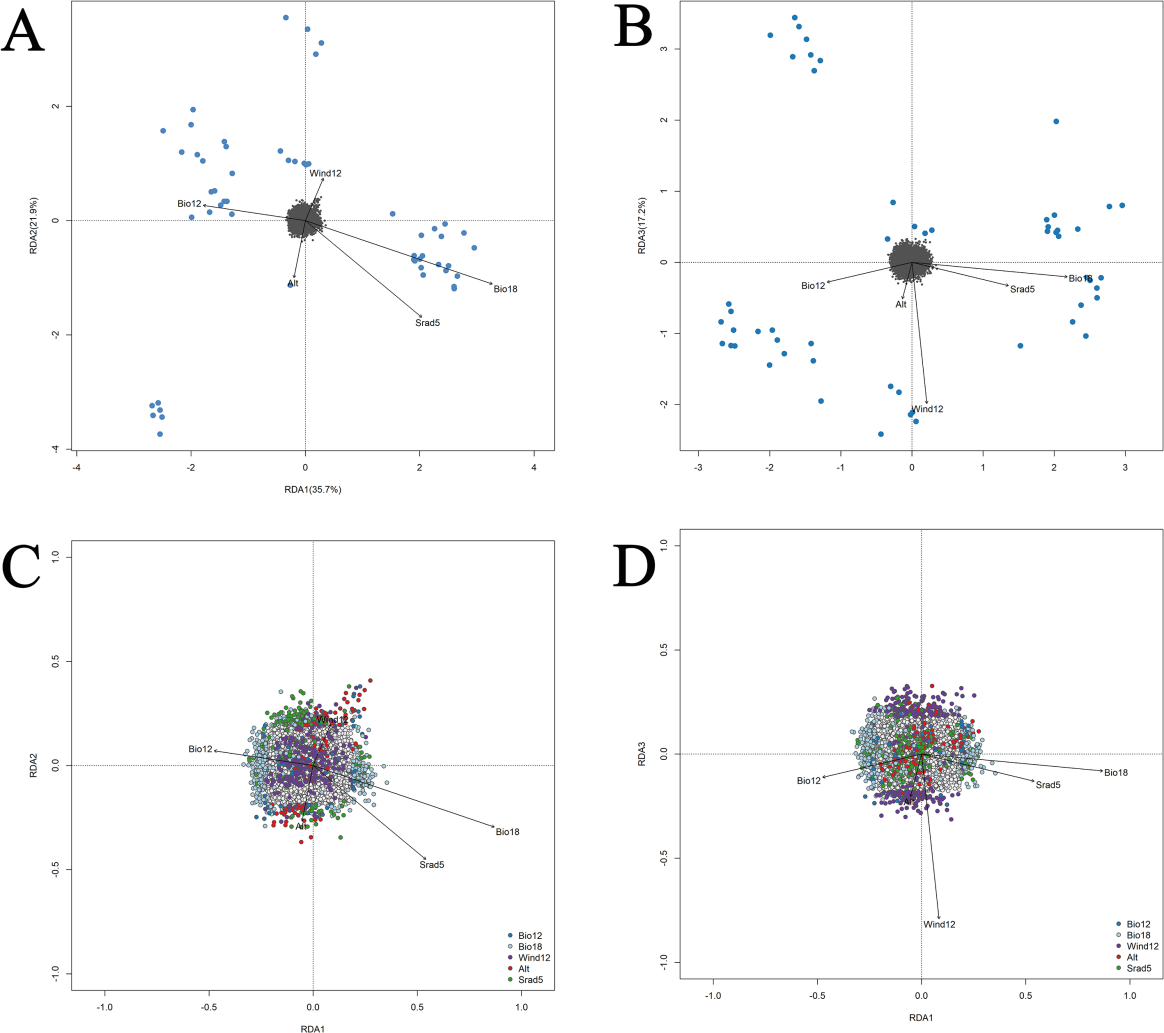
Redundancy analysis (RDA) plot for the 54 *Picea asperata* individuals. **(A)** Redundancy Analysis (RDA) plot based on RDA1 and RDA2 axes. **(B)** RDA plot based on RDA1 and RDA3 axes. **(C)** Zoomed-in view of plot A, highlighting the loading distribution of SNP on RDA1 and RDA2 axes. **(D)** Zoomed-in view of plot B, highlighting the SNP loadings on RDA1 and RDA3 axes.

Redundancy analysis (RDA) revealed strong explanatory power of environmental factors on the genomic differentiation of *P. asperata*. Specifically, RDA1, RDA2, and RDA3 accounted for 35.7%, 21.9%, and 17.2% of the variation, respectively, with the first three axes cumulatively explaining 74.8% of the total variation (**Figure 4**). Environmental factors exhibited significant constraints on the genomic differentiation (F = 1.2752, *p* < 0.001), confirming their robust explanatory capacity. From an initial pool of 2,104 candidate environment-associated SNPs, 2,030 were retained after deduplication, which exhibited specific correlation distributions with various environmental variables. The highest number of associated SNPs was found for Wind12 (n = 656, 32.3%), followed by Bio18 (n = 587, 28.9%), Srad5 (n = 451), Alt (n = 224), and Bio12 (n = 112). Latent factor mixed models (LFMM) detected 1,113 SNPs associated with at least one environmental variable. The number of SNPs associated with each environmental factor was 589 for Bio18, 581 for Alt, 302 for Bio12, 292 for Srad5, and 39 for Wind12. Comparison of the two methods revealed 64 SNPs commonly identified by both RDA and LFMM (**Supplementary Figure 3**), representing high-confidence candidates for local adaptation.

GO enrichment analysis of the 64 shared SNPs revealed several significantly enriched terms, including “cytoplasm” (GO:0005737), “nucleus” (GO:0005634), “plasmodesma” (GO:0009506), and “DNA−templated transcription” (GO:0006351) (**Supplementary Figure 4**). Functional annotation by BLAST against the *Arabidopsis thaliana* proteome identified the best−hit proteins and corresponding functions (**Supplementary Table 5**). Among these, three were directly related to environmental stress responses: Early responsive to dehydration 1 (*ERD1*), *Arabidopsis* response regulator 1 (*ARR1*), and Indole-3-butyric acid response 1 (*IBR1*).

## 4 Discussion

### 4.1 Relatively low genetic diversity of *Picea asperata*

ADMIXTURE analysis indicated that the optimal number of clusters was K = 1 (**Supplementary Table 1**). *P. asperata* demonstrates a *π* value of 0.003508, which is similar to previous reported values (Bi et al., 2016; Feng et al., 2023a, Feng et al., 2025). Comparative analyses of other *Picea* species, including *P. abies*, *P. wilsonii*, *P. neoveitchii*, *P. purpurea*, and *P. likiangensis*, maintain higher genetic diversity, with *π* values ranging from 0.0039 to 0.00691 (Zou et al., 2013; Ru et al., 2018; Wang et al., 2020), suggesting lower genetic diversity in *P. asperata*. Pairwise *F*_ST_ values among all populations were low (**Supplementary Table 3**), indicating weak genetic differentiation. TreeMix analysis further revealed ongoing gene flow (**Figures 2A, B**). This result implies that contemporary gene flow, likely via pollen or seed dispersal, contributes to the homogenization of genetic variation across populations.

We detected a pronounced bottleneck in *P. asperata* using Stairway Plot analysis (**Figure 2C**), suggesting that the species has experienced a historical population contraction followed by subsequent expansion. This demographic inference is further supported by the positive Tajima’s *D* value (**Table 2**) obtained in our study. In population genetics, a positive Tajima’s *D* typically indicates an excess of intermediate-frequency alleles and a deficiency of rare alleles, a pattern characteristic of populations that have undergone a recent bottleneck or contraction. Such demographic dynamics often lead to population size contraction during the bottleneck effect, followed by expansion, thereby shaping the standing genetic variation. The timing of this bottleneck corresponds to a period when Quaternary glaciations intensified and the global climate began oscillating between ice ages and interglacials. Repeated climate oscillations during this period drove cycles of habitat contraction and expansion for temperate plant species (Qiu et al, 2011; Liu et al, 2012). Such contractions often led to bottlenecks and founder effects that reduced effective population sizes, leaving lasting imprints on genetic diversity. For *P. asperata* on the Qinghai-Tibet Plateau, these climatic fluctuations likely forced populations to retreat into isolated refugia during glacial maxima. Studies have proposed that microrefugia existed on the plateau at elevations above 3,500 m during the Quaternary maximum glaciation, providing critical shelter for forest species (Opgenoorth et al., 2010). Following deglaciation, populations may have expanded from these refugia. Taken together, the low overall genetic diversity in *P. asperata* is likely attributable to a historical population bottleneck shaped by Quaternary climate oscillations.

### 4.2 The impact of environmental factors on genomic differentiation

Based on redundancy analysis (RDA), we can determine that the environmental variables will have an effect on the genetic divergence of *P. asperata*. Five key environmental variables were found to be important: December wind speed (Wind12), precipitation of the warmest quarter (Bio18), May solar radiation (Srad5), altitude (Alt), and annual precipitation (Bio12). The cumulative proportion of variance explained by RDA1, RDA2, and RDA3 was 74.8%, indicating that these environmental factors have a strong explanation of population genetic structure. To further validate and refine these associations, we complemented the RDA with LFMM. LFMM identified 1,113 SNPs associated with environmental variables. The overlap between RDA and LFMM results identified 64 SNPs.

Among RDA, December wind speed (Wind12) and precipitation of warmest quarter (Bio18) were associated with 656 and 587 SNP loci, respectively. December wind speed (Wind12) significantly affects the overwinter survival rate of *P. asperata* seedlings by regulating winter transpiration in branches. This persistent selective pressure not only directly influences population growth stability and reproductive dispersal efficiency but also drives genomic differentiation by constraining key adaptive traits related to winter water management (Juergen et al., 2014; McIntire et al., 2016). Precipitation of the warmest quarter (Bio18) influences wood formation processes by modulating water availability during the growing season. This sustained environmental selection acts on individuals’ growth rates, serving as a key driver of population genetic differentiation (Marano et al., 2024).

To further elucidate the biological significance of these 64 consensus SNPs, we performed functional annotation and Gene Ontology (GO) enrichment analysis. BLAST analysis against the *Arabidopsis thaliana* database identified high-confidence homologs directly involved in environmental adaptation and stress responses. Notably, we identified a homolog of *ERD1*, a gene essential for drought tolerance and early response to water deprivation. Additionally, we identified *ARR1*, a key transcription factor involved in cytokinin signaling and abiotic stress responses, and *IBR1*, which is linked to auxin metabolism and environmental stimulus response (**Supplementary Table 5**).

Based on these findings, we recommend region-specific conservation and afforestation strategies that account for local environmental conditions. By selecting germplasm resources with genetic traits adapted to dominant environmental factors in each region, scientifically guided breeding can enhance genotype-environment matching, thereby effectively improving the genetic diversity of *P. asperata*.

### 4.3 Conservation strategies under rapid climate change

Based on the projections of MaxEnt modeling, it can be seen that the current distribution of *P. asperata* is close to the predicted suitable habitats, especially high-suitability areas. Future projections under both SSP1−2.6 and SSP5−8.5 scenarios reveal a consistent trend of initial habitat expansion followed by contraction, with more pronounced fluctuations under the high−emission SSP5−8.5 pathway. Throughout both scenarios, the total suitable area remains larger than the present distributions. The projected initial expansion phase likely stems from CO_2_-enhanced photosynthesis and extended growth ranges under moderate warming, while subsequent contraction reflects temperature thresholds being exceeded and increased drought-induced habitat degradation. These dynamics represent the integrated effects of rising temperatures, shifting precipitation patterns, elevated atmospheric CO_2_ (Ray et al., 2025), and more frequent extreme climate events. Similar distribution patterns have been documented in the endemic Qinghai-Tibet Plateau species *P. likiangensis* and *P. purpurea*, which also exhibit initial expansion-contraction cycles while maintaining larger future suitable areas than current ranges (Sun et al., 2014a; Sun et al., 2014b).

Considering that the limited genetic diversity of *P. asperata* may restrict its climate adaptability, we propose an integrated conservation approach. The integration of GEA and MaxEnt results provides a more comprehensive basis for conservation prioritization. Priority should be given to conserving germplasm resources that carry key adaptive alleles and to assisted migration of such germplasm to areas projected to remain suitable under future climates. Establishing comprehensive germplasm repositories should be prioritized alongside selective and hybrid breeding programs for genetic enhancement. Concurrently, developing advanced seedling cultivation systems will support climate−adaptive afforestation efforts. Priority should also be given to maintaining existing habitat connectivity and avoiding future anthropogenic fragmentation, which is essential for preserving genetic connectivity among populations, particularly given evidence that interspecific introgression within *Picea* and related taxa provides critical genetic foundations for environmental adaptation (Oziolor et al., 2019; Brauer et al., 2023; Satokangas et al., 2024; Feng et al., 2025; Geng et al., 2025). Proactive measures should also include assisted migration of germplasm into projected future suitable habitats to bolster the species’ adaptive capacity. This multifaceted strategy simultaneously addresses population viability and genetic diversity conservation in response to climate change challenges.

## 5 Conclusion

This study employed transcriptome sequencing of 54 individuals from 11 populations of *P. asperata* to analyze its population structure, genetic diversity, and climate adaptation. ADMIXTURE analysis indicated an optimal clustering number of K = 1, and the species exhibited low genetic diversity at the species level (*π* = 0.003508). Stairway Plot analysis detected a historical bottleneck followed by gradual population recovery. GEA analyses, including RDA and LFMM, identified December wind speed (Wind12) and precipitation of the warmest quarter (Bio18) as key environmental drivers of genomic differentiation. Functional annotation and GO enrichment of the 64 shared SNPs uncovered candidate genes and biological processes associated with environmental adaptation. MaxEnt projections indicate that the species’ suitable habitat will expand under future climate scenarios, especially under SSP5−8.5. To safeguard the adaptive potential of *P. asperata*, we recommend strengthening *in situ* conservation, maintaining existing habitat connectivity, assisting migration of germplasm carrying key adaptive alleles to areas projected to remain suitable under future climates, and establishing comprehensive germplasm repositories. These findings provide a scientific basis for the conservation of *P. asperata* and contribute to understanding species adaptability under future climate change.

## Data Availability

The raw data generated in this study can be found in the NCBI repository (accession PRJNA846694) and is publicly accessible at the following link: https://www.ncbi.nlm.nih.gov/bioproject/PRJNA846694.
