# Supplementary material for: Genetic diversity and distribution pattern responses of Picea asperata
Source: Front Plant Sci. 2026 May 28;17:1808288. doi: 10.3389/fpls.2026.1808288 (PMC13253503; doi:10.3389/fpls.2026.1808288)
Supplement: Supplementary file 1 [file DataSheet1.docx]

Supplementary Material

**Supplementary TABLE 1.** Environmental variables in this study.

| Name | Environmental variable |
| --- | --- |
| Bio1 | Annual Mean Temperature/℃ |
| Bio2 | Mean Diurnal Range/℃ |
| Bio3 | Isothermality (Bio2/Bio7) (×100) |
| Bio4 | Temperature Seasonality (standard deviation×100) |
| Bio5 | Max Temperature of Warmest Month/℃ |
| Bio6 | Min Temperature of Coldest Month/℃ |
| Bio7 | Temperature Annual Range (Bio5-Bio6)/℃ |
| Bio8 | Mean Temperature of Wettest Quarter/℃ |
| Bio9 | Mean Temperature of Driest Quarter/℃ |
| Bio10 | Mean Temperature of Warmest Quarter/℃ |
| Bio11 | Mean Temperature of Coldest Quarter/℃ |
| Bio12 | Annual Precipitation/mm |
| Bio13 | Precipitation of Wettest Month/mm |
| Bio14 | Precipitation of Driest Month/mm |
| Bio15 | Precipitation Seasonality (Coefficient of Variation)/mm |
| Bio16 | Precipitation of Wettest Quarter/mm |
| Bio17 | Precipitation of Driest Quarter/mm |
| Bio18 | Precipitation of Warmest Quarter/mm |
| Bio19 | Precipitation of Coldest Quarter/mm |
| Alt | Altitude/m |
| Srad1 - 12 | Solar Radiation from January to December/(kJ·m^-2^·d^-1^) |
| Wind1 - 12 | Wind Speed from January to December/(m·s^-1^) |
| Vapr1 - 12 | Water Vapor Pressure from January to December/kPa |
| Prec1 - 12 | Precipitation from January to December/mm |

**Supplementary TABLE 2.** Overview of sample information and sequencing statistics.

| Samples | Covered Sites | Sum Depth | Genome Coverage | Effective Depth | Map Ratio |
| --- | --- | --- | --- | --- | --- |
| SRR19571789 | 43,934,529 | 2,369,804,307 | 0.64 | 34.40 | 0.59 |
| SRR19571800 | 45,836,242 | 3,061,159,582 | 0.67 | 44.44 | 0.63 |
| SRR19571811 | 44,998,256 | 3,209,654,276 | 0.65 | 46.59 | 0.63 |
| SRR19571879 | 45,729,658 | 2,184,197,803 | 0.66 | 31.71 | 0.63 |
| SRR19571881 | 46,745,654 | 2,539,644,223 | 0.68 | 36.87 | 0.65 |
| SRR19571882 | 47,442,074 | 2,542,838,589 | 0.69 | 36.91 | 0.65 |
| SRR19571883 | 48,743,331 | 2,631,545,908 | 0.71 | 38.20 | 0.65 |
| SRR19571884 | 48,322,213 | 2,573,063,863 | 0.70 | 37.35 | 0.65 |
| SRR19571900 | 46,713,730 | 2,330,131,919 | 0.68 | 33.82 | 0.61 |
| SRR19571903 | 46,097,042 | 2,382,952,320 | 0.67 | 34.59 | 0.62 |
| SRR19571906 | 45,562,016 | 2,384,913,145 | 0.66 | 34.62 | 0.63 |
| SRR19571907 | 45,852,920 | 2,500,625,433 | 0.67 | 36.30 | 0.63 |
| SRR19571909 | 44,706,150 | 2,346,829,505 | 0.65 | 34.07 | 0.60 |
| SRR19571985 | 48,523,417 | 2,727,598,784 | 0.70 | 39.59 | 0.66 |
| SRR19571986 | 48,693,330 | 2,756,282,432 | 0.71 | 40.01 | 0.66 |
| SRR19571994 | 47,452,150 | 2,882,233,428 | 0.69 | 41.84 | 0.68 |
| SRR19571997 | 46,862,531 | 2,824,261,232 | 0.68 | 41.00 | 0.66 |
| SRR19571998 | 48,099,676 | 2,843,363,463 | 0.70 | 41.28 | 0.67 |
| SRR19571999 | 48,514,034 | 2,921,791,841 | 0.70 | 42.41 | 0.66 |
| SRR19572000 | 49,505,680 | 3,366,749,417 | 0.72 | 48.87 | 0.65 |
| SRR19572001 | 47,857,451 | 2,679,819,501 | 0.69 | 38.90 | 0.65 |
| SRR19572002 | 47,006,619 | 2,586,722,955 | 0.68 | 37.55 | 0.65 |
| SRR19572003 | 46,004,135 | 2,715,894,714 | 0.67 | 39.42 | 0.67 |
| SRR19572004 | 48,983,334 | 2,782,298,662 | 0.71 | 40.39 | 0.67 |
| SRR19572008 | 48,003,791 | 2,717,946,410 | 0.70 | 39.45 | 0.67 |
| SRR19572009 | 45,764,189 | 2,542,383,458 | 0.66 | 36.91 | 0.68 |
| SRR19572010 | 44,256,997 | 2,773,273,884 | 0.64 | 40.26 | 0.70 |
| SRR19572012 | 44,135,324 | 2,989,025,494 | 0.64 | 43.39 | 0.69 |
| SRR19572014 | 41,118,334 | 2,550,118,607 | 0.60 | 37.02 | 0.70 |
| SRR19572054 | 48,418,359 | 2,666,297,172 | 0.70 | 38.70 | 0.65 |
| SRR19572056 | 44,038,391 | 2,613,892,287 | 0.64 | 37.94 | 0.64 |
| SRR19572058 | 47,311,651 | 2,714,412,187 | 0.69 | 39.40 | 0.65 |
| SRR19572060 | 45,091,312 | 2,964,433,952 | 0.65 | 43.03 | 0.64 |
| SRR19572062 | 46,017,637 | 2,586,694,455 | 0.67 | 37.55 | 0.67 |
| SRR19572065 | 48,610,287 | 2,928,046,384 | 0.71 | 42.50 | 0.67 |
| SRR19572066 | 48,948,754 | 2,866,789,906 | 0.71 | 41.62 | 0.66 |
| SRR19572067 | 47,347,638 | 2,552,591,410 | 0.69 | 37.05 | 0.65 |
| SRR19572068 | 46,357,302 | 2,846,356,446 | 0.67 | 41.32 | 0.66 |
| SRR19572069 | 47,265,198 | 2,829,194,687 | 0.69 | 41.07 | 0.65 |
| SRR19572071 | 42,799,143 | 2,805,616,323 | 0.62 | 40.73 | 0.66 |
| SRR19572077 | 47,789,641 | 2,560,806,394 | 0.69 | 37.17 | 0.66 |
| SRR19572078 | 46,981,018 | 2,596,431,984 | 0.68 | 37.69 | 0.64 |
| SRR19572079 | 47,242,058 | 2,539,584,602 | 0.69 | 36.87 | 0.65 |
| SRR19572080 | 47,854,875 | 2,621,596,619 | 0.69 | 38.06 | 0.65 |
| SRR19572081 | 48,146,125 | 2,447,937,640 | 0.70 | 35.54 | 0.65 |
| SRR19572082 | 44,274,993 | 3,059,013,727 | 0.64 | 44.41 | 0.62 |
| SRR19572084 | 48,230,774 | 2,745,386,316 | 0.70 | 39.85 | 0.65 |
| SRR19572111 | 49,275,407 | 2,816,261,976 | 0.72 | 40.88 | 0.66 |
| SRR19572113 | 43,656,266 | 2,764,481,753 | 0.63 | 40.13 | 0.65 |
| SRR19572114 | 48,592,840 | 2,871,519,435 | 0.71 | 41.68 | 0.66 |
| SRR19572118 | 47,891,367 | 2,806,403,766 | 0.70 | 40.74 | 0.64 |
| SRR19572119 | 47,631,487 | 2,943,619,880 | 0.69 | 42.73 | 0.66 |
| SRR19572120 | 47,433,208 | 2,653,489,082 | 0.69 | 38.52 | 0.66 |
| SRR19572121 | 47,962,930 | 2,905,026,777 | 0.70 | 42.17 | 0.66 |
| Mean | 46,789,509 | 2,711,537,228 | 1.00 | 39.00 | 1.00 |

**Supplementary TABLE 3.** Pairwise genetic differentiation (*F*_ST_) matrix between *Picea asperata* populations.

| *F*_ST_ | Asp01 | Asp02 | Asp03 | Asp04 | Asp05 | Asp06 | Asp07 | Asp08 | Asp09 | Asp11 | Asp12 |
| --- | --- | --- | --- | --- | --- | --- | --- | --- | --- | --- | --- |
| Asp01 |  | 0.014289 | 0.005756 | 0.024519 | 0.017424 | 0.018547 | 0.035265 | 0.050765 | 0.056779 | 0.053155 | 0.014477 |
| Asp02 |  |  | 0.004491 | 0.029906 | 0.024594 | 0.031909 | 0.048067 | 0.057394 | 0.059480 | 0.008992 | 0.009713 |
| Asp03 |  |  |  | 0.018349 | 0.013875 | 0.020231 | 0.038347 | 0.039105 | 0.042776 | 0.053269 | 0.001576 |
| Asp04 |  |  |  |  | 0.004833 | 0.009956 | 0.021757 | 0.024890 | 0.027145 | 0.068003 | 0.016875 |
| Asp05 |  |  |  |  |  | 0.007798 | 0.023160 | 0.021347 | 0.028554 | 0.048412 | 0.012282 |
| Asp06 |  |  |  |  |  |  | 0.014707 | 0.012134 | 0.016039 | 0.046246 | 0.018825 |
| Asp07 |  |  |  |  |  |  |  | 0.015359 | 0.015976 | 0.051790 | 0.036904 |
| Asp08 |  |  |  |  |  |  |  |  | 0.003442 | 0.079049 | 0.040626 |
| Asp09 |  |  |  |  |  |  |  |  |  | 0.083575 | 0.048632 |
| Asp11 |  |  |  |  |  |  |  |  |  |  | 0.070942 |
| Asp12 |  |  |  |  |  |  |  |  |  |  |  |

**Supplementary TABLE 4.** Genetic diversity parameters in *Picea asperata*.

| Parameter | *H*_e_ | *H*_o_ |
| --- | --- | --- |
| *Picea asperata* | 0.215927 | 0.306883 |

**Supplementary TABLE 5.** Environmentally relevant best-hit proteins and corresponding functions identified by BLAST search against the *Arabidopsis thaliana* proteome.

| ID | Locus | Name | Description |
| --- | --- | --- | --- |
| comp82049_c1_seq1 | AT5G51070.1 | *ERD1* / *SAG15* / *CLPD* | Early responsive to dehydration 1, senescence-associated gene 15; involved in drought and dehydration stress |
| comp83963_c0_seq1 | AT3G16857.1 | *ARR1* / *RR1* | Response regulator 1; involved in cytokinin signaling and stress-responsive gene expression |
| comp97689_c0_seq1 | AT4G05530.1 | *IBR1* / *SDRA* | Indole-3-butyric acid response 1, short-chain dehydrogenase / reductase A; involved in auxin metabolism and oxidative stress response |


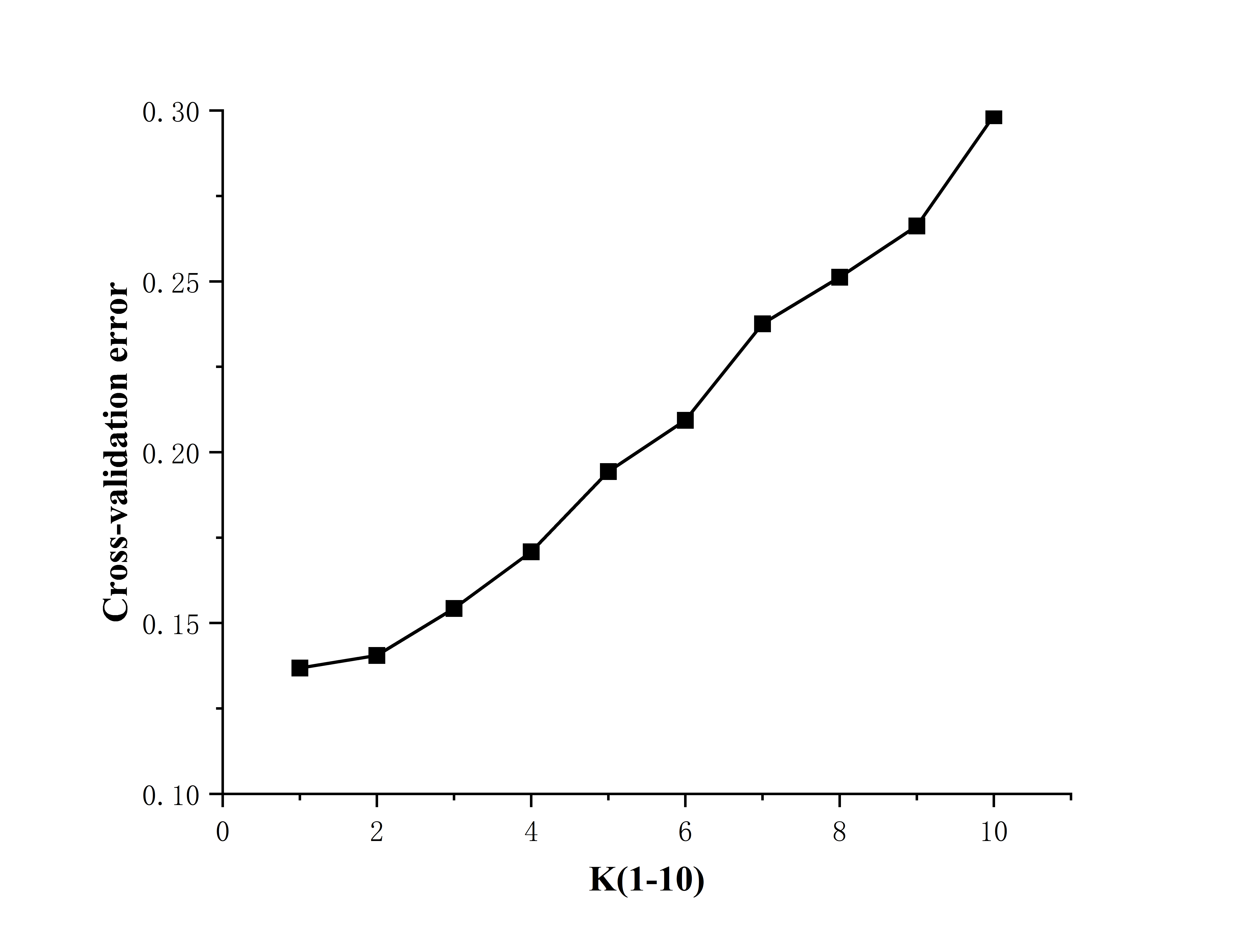


**Supplementary FIGURE 1.** Cross-validation (CV) error value for different model K**.**

**
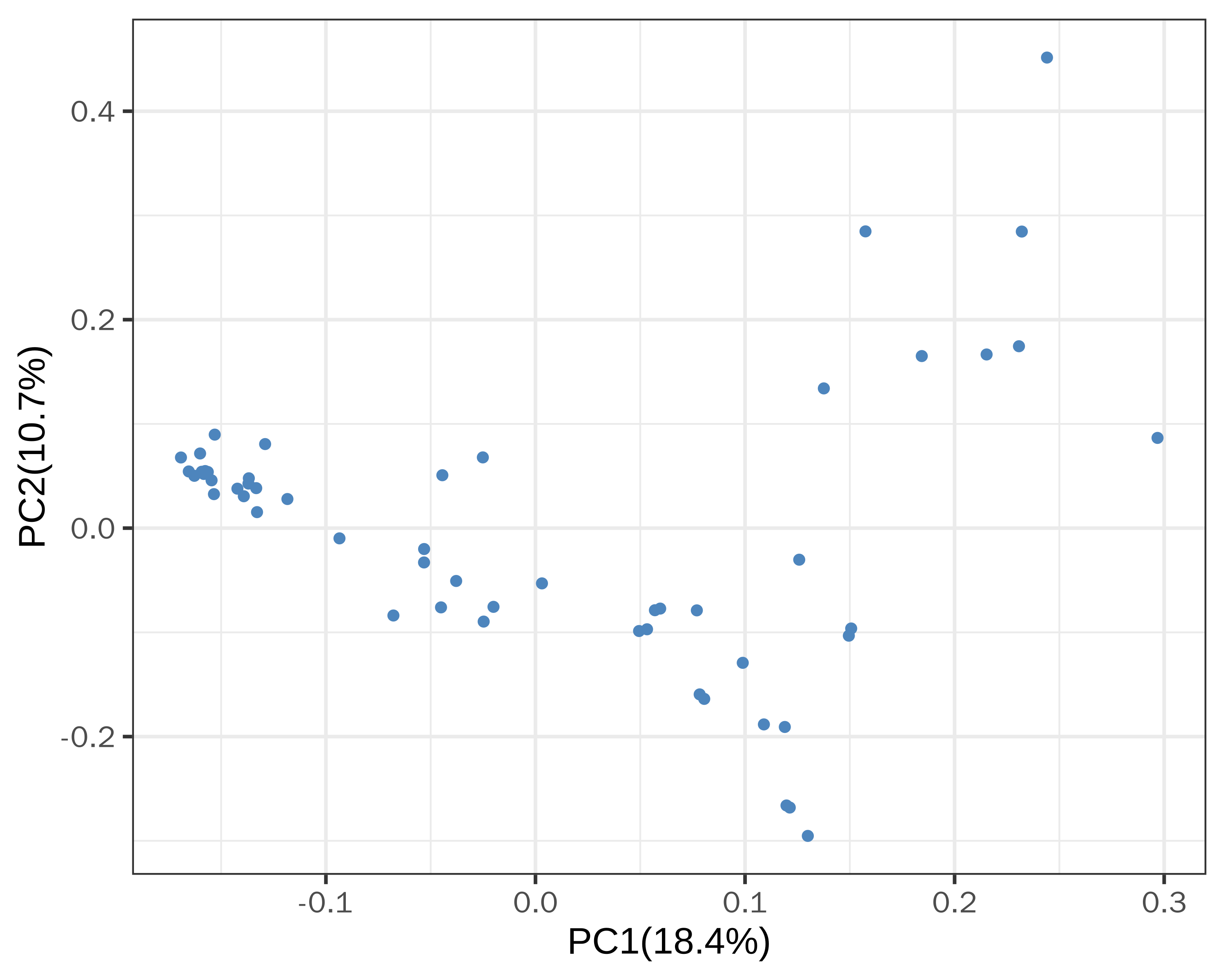
Supplementary FIGURE 2.** Principal component analysis biplot of genetic variants in *Picea asperata*.


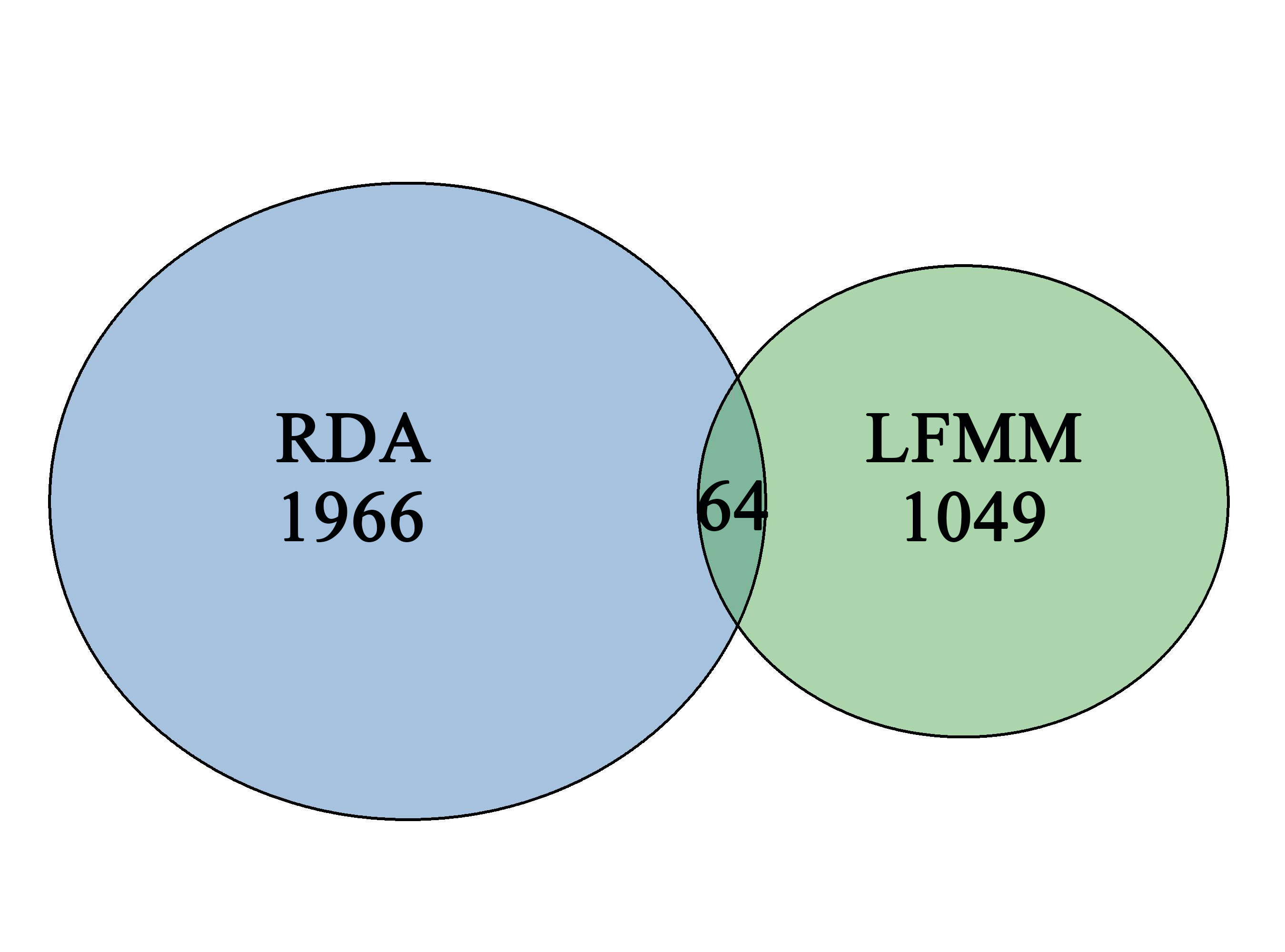
**Supplementary FIGURE 3.** Venn of environment-associated SNPs identified by RDA and LFMM methods.


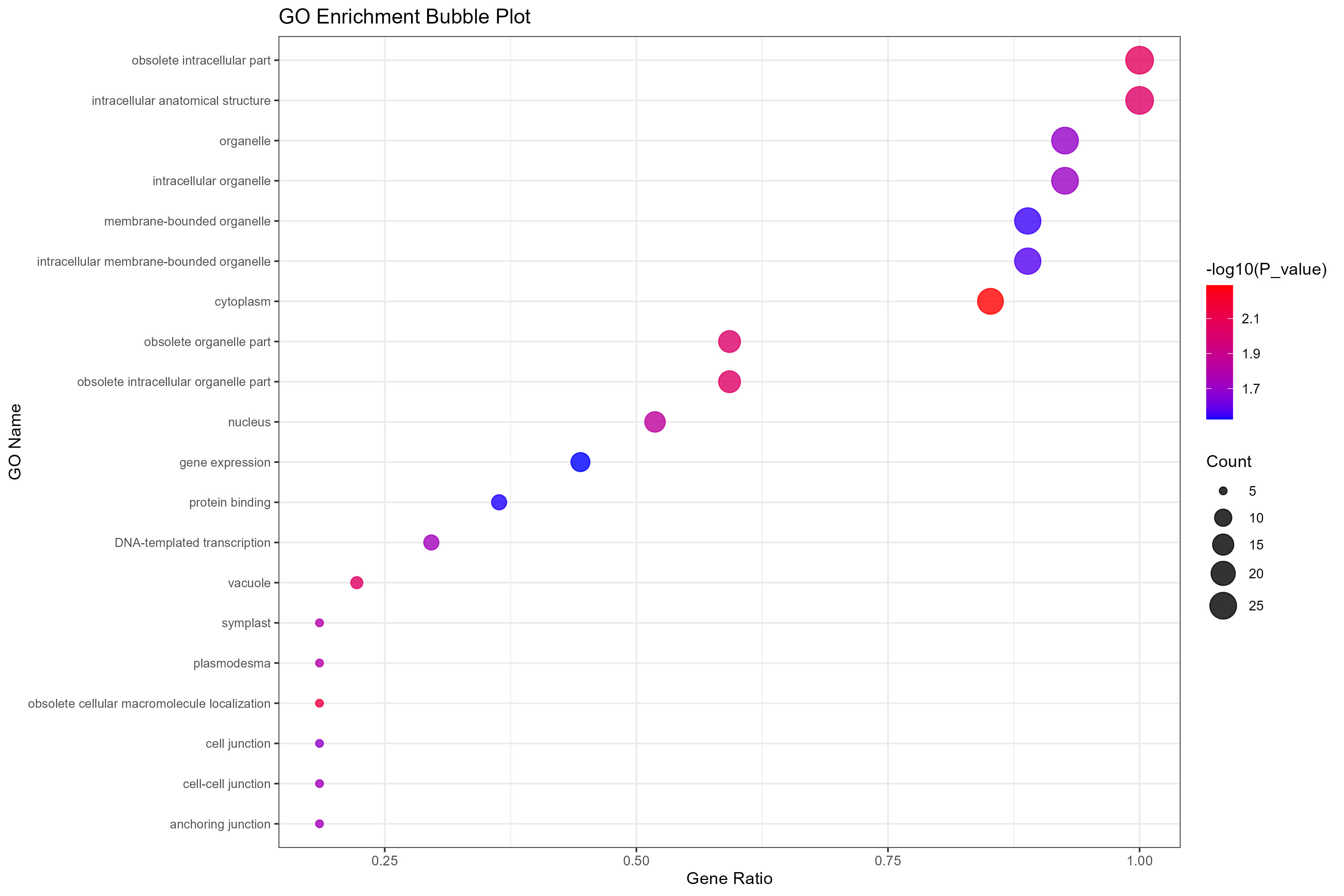


**Supplementary FIGURE 4.** GO enrichment bubble plot.
